# Two innovative Brazilian programs relating to road safety prevention. A case study

**DOI:** 10.1590/1516-3180.2019.137150319lpc

**Published:** 2019-09-05

**Authors:** Leandro Piquet Carneiro, Linamara Rizzo Battistella

**Affiliations:** I MSc, PhD. Economist and Professor, International Relations Institute, Universidade de São Paulo (USP), São Paulo (SP), Brazil.; II MD, PhD. Professor, Department of Legal Medicine, Medical Ethics and Social and Labor Medicine, Faculdade de Medicina da Universidade de São Paulo (FMUSP), São Paulo (SP), Brazil.

**Keywords:** Noncommunicable disease, Alcohol drinking, Public-private sector partnerships

## Abstract

**BACKGROUND:**

The World Health Organization (WHO) 2017 Global Conference in Montevideo, Uruguay, was dedicated to promoting successful cases and best practices in fighting and preventing noncommunicable disease (NCDs). The global effort undertaken by WHO aims to reduce road traffic deaths in order to meet goal number 3.4 of the sustainable development goals.

**OBJECTIVES:**

To describe two Brazilian road safety prevention programs, presented at the WHO 2017 Global Conference: São Paulo Traffic Safety Movement (Movimento Paulista de Segurança no Trânsito) and Safe Life Program of Brasília (Programa Brasília Vida Segura), along with their governance structures, models and results.

**DESIGN AND SETTING:**

This was a descriptive case study conducted in São Paulo and Brasilia from 2015 to 2018. These programs aimed to reduce the number of deaths caused by road accidents to 8.3 deaths per 100,000 inhabitants in São Paulo by 2020 and in Brasília by 2016; and to reduce harmful use of alcohol by 10% by 2020.

**METHODS:**

These two initiatives were designed, managed and operated to bring together government and civil society, i.e. industry, academia, non-governmental organizations (NGOs), etc., around the common goal of saving lives. They were collaborative and guided by sharing of best practices, learning and information, thereby making it possible to attain more and better results. Their format enables reproduction in cities across all Brazilian regions.

**RESULTS:**

The results attest to the efficacy of the programs implemented in these two cities. In Brasília, the initiative helped reduce the number of traffic-related deaths by 35% (2017). In the same year in the state of São Paulo, 7,600 deaths were avoided.

**CONCLUSION:**

Both programs are innovative public policies that deal with health issues caused by the external agents that ultimately account for the rapid increase in days lost to disability. Prevention of external causes of deaths and injuries, such as traffic violence, strongly correlates with changes in habits and actions, especially excessive consumption of alcohol, and with NCDs in Brazil.

## INTRODUCTION

Noncommunicable diseases (NCDs) are currently one of the greatest challenges faced within public health. Their impact is so severe that the World Health Organization (WHO) has come to classify them as an epidemic. According to the organization, around 36 million people die prematurely due to cardiovascular and respiratory diseases, cancer and diabetes worldwide every year.^1^ Around 85% of the deaths caused by NCDs occur either in poor or in developing countries, which contributes towards further aggravation of the situation^1^. The main risk factors are excessive alcohol consumption, smoking, unbalanced diets and lack of exercise.^1^

Just as drinking too much alcohol is a risk factor for NCDs, the association of drinking and driving is one of the main causes of traffic accidents.^1^ According to a study conducted by the World Health Organization, approximately 1.25 million people die in road accidents around the world every year, and another 50 million get injured.^1^ Aside from natural causes of death, traffic accidents are one of the leading causes of death among young people between 15 and 29 years of age, at the global level.^1^ If this upward trend is not reversed, deaths due to traffic accidents may become the seventh biggest reason for loss of life worldwide by 2030.^2^ This would represent a mortality rate 30% higher than the 10% rate recorded in 2015.^2^ To tackle this challenge, raise awareness and mobilize the global community, WHO has defined the period between 2011 and 2020 as the decade of action for traffic safety. The goal is to halve the number of accidents and save five million lives.^2^

Traffic accidents can cause chronic or permanent physical or mental damage. These effects require lifelong care for the individuals involved and will often prevent them from having a normal routine or from working. The resultant morbidity can be understood as a noncommunicable disease (NCD). In speaking about NCDs, external causes such as accidents should be thought of as factors that are just as important as cardiovascular and respiratory factors. The social impact of these occurrences arises from the cost of disability-adjusted life years. According to the Global Burden of Disease Study (2015),^2^ 0.32% of all deaths worldwide (around 54.7 million) are caused by disorders due to harmful use of alcohol. In Brazil, this percentage hits 0.70%, which corresponds to 1.28 million lives lost.^3^ The number of disability-adjusted life years relating to disorders caused by harmful use of alcohol reaches 1.25% at the global level (around 805.4 million cases) and 2.64% (about 22 million) in Brazil. The leading cohort in the ranking of deaths is the one composed of stroke victims, which accounts for 4.92% of all deaths worldwide and 5.1% in Brazil.^3^

## OBJECTIVE

To describe two innovative Brazilian programs relating to noncommunicable diseases that were presented at the World Health Organization (WHO) 2017 Global Conference.

## METHODS

This was a descriptive case study conducted in São Paulo in 2015 about two programs aimed to reduce the number of deaths caused by road accidents to 8.3 deaths per 100,000 inhabitants in São Paulo by 2020 and in Brasília by 2016; and to reduce harmful use of alcohol by 10% by 2020. The two programs were named the São Paulo Traffic Safety Movement (Movimento Paulista de Segurança no Trânsito) and Safe Life Program of Brasília (Programa Brasília Vida Segura). They were presented at the Global Conference on NCDs and targeted the two challenges that WHO had proposed: prevention and combating of noncommunicable diseases and reduction of traffic-related deaths.^2^

These two initiatives were designed, managed and operated to bring together government and civil society, i.e. industry, academia, non-governmental organizations (NGOs), etc., around the common goal of saving lives. They were collaborative and guided by sharing of best practices, learning and information, thereby making it possible to attain more and better results. Their format enables reproduction in cities across all Brazilian regions.

### Internal review board

The data collection conducted in Brazil (the Federal District) is part of a research initiative carried out in partnership with the Pacific Institute for Research and Evaluation (PIRE). The study protocol was duly evaluated and approved by an Internal Review Board from the North American institution (IRBNET ID: 1116187-18; approval date: August 28, 2018).

## RESULTS

The application of the model yielded surprising results that went beyond reduction of deaths and injuries in traffic accidents. Educational actions that were developed in schools and public spaces made the programs more sustainable. These interventions highlighted the positive effects of tackling harmful use of alcohol and the association of risks between drinking and driving, among other related topics.

### São Paulo Traffic Safety Movement (Movimento Paulista de Segurança no Trânsito)

The São Paulo Traffic Safety Movement was launched in 2015 and is an example of a result-oriented public policy aimed towards preventing and combating NCDs. It has very strong educational leanings and can be understood as a pioneering initiative for combating chronic noncommunicable diseases. Its actions, developed in an integrated manner, are aimed at promotion of road safety as a whole. Its implementation includes information, awareness and oversight vectors.

This project was conceived as a coalition between the public sector, private sector, academia and around 20 entities within civil society. It reflects the commitment of the state of São Paulo towards addressing NCDs. To this end, the state involved nine government departments, which shared information and acted in tandem. This movement made it possible for actions that were being developed separately or that were scattered among various state departments, to start to be analyzed as a group, in a way that was result-driven. This meant efforts were synchronized so that everyone could work towards the same goal: to reach the objective established by WHO.

The São Paulo Traffic Safety Movement has taken “make way for life” (*vida, dê preferencia*) as its motto and its aim is to reduce the number of deaths caused by road accidents to 8.3 deaths per 100,000 inhabitants in São Paulo by 2020.

In implementing this movement, 15 cities were initially prioritized. The state capital (city of São Paulo) was one of them. In total, 122 actions were identified, and these were divided into three types: engineering, education and communication plus control. Each of the municipalities thus selected received a customized action plan, so that the program could be implemented quickly and effectively.

The implementation of the program evolved rapidly, and all 645 municipalities in São Paulo are now part of the movement. Table 1 shows details regarding the items corresponding to each of the three types of action.

**Table 1.** Actions: at the right place and time.

Engineering	Installing footbridges	Installing traffic lights	Bicycle paths	Lowering speed limit
Education and Communication	Training public traffic agents	Traffic education campaigns	Creating a road safety website	Supporting the mobility of bicycles and pedestrians
Control	Surveilling critical spots			

Source: Evidence-based public policies for combating NCDs. Presented by Battistella (2018) at the WHO Global Conference on NCDs (Montevideo, 2018).

**Figure 1.**
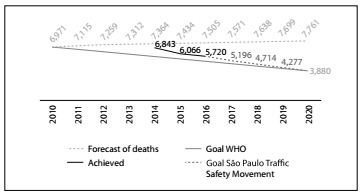
Projection of traffic deaths and reduction goal: state of São Paulo.

The first results from the movement have shown that the design and dynamics of the program are on track to achieve the stated goal. In 2016, 460 lives were saved, in the 15 municipalities that were initially chosen to receive the program. This number represented 6.5% fewer deaths than in 2015. In the following year (2017), an astounding 7,600 casualties were avoided. The aim of the movement now is to get to 2020 with a maximum of 3,880 accidents involving victims. If nothing were to be done, this number would double, reaching 7,760 accidents. Figure 1 shows the expected trend and the goals set by the project year by year.

The conception of the São Paulo Traffic Safety Movement considered that the challenges proposed by WHO to address the growth of NCDs required a collaborative model. Thus, the coalition and the combination of efforts of different public, private and civil society agents was created based on this very principle.

The private sector contributed the resources needed for implementation and management of the movement, shared knowledge about process management and brought the project into a result-driven culture. It also invested the energy of its team in training professionals and mobilizing communities that would be benefited through the project. The state of São Paulo has made a political commitment to make public agents available and to link them together (government departments, the police, institutions relating to healthcare and education, traffic agencies, etc.) for actions aimed towards preventing and combating excessive consumption of alcoholic beverages and its association with driving.

**Figure 2.**
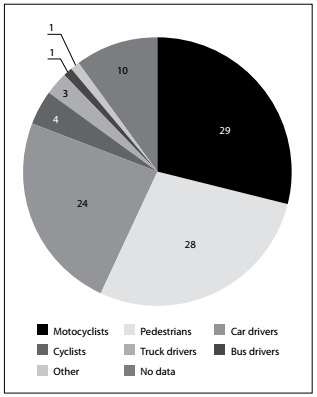
Deaths: users and vehicles (%).

Through communication and engagement actions, civil society agents contributed towards dissemination of information and guidance to the population, thereby multiplying the impact of the project. This whole process used a method developed by Falconi, the consulting firm that was in partnership with the project.^4^ Defining a working method makes it possible to implement the project in any municipality. The flexibility of the model thus developed allowed it to be adapted to the characteristics of each location, thereby maintaining its principles of seeking consistent results that met the goals that had been established. Figure 2 shows how the governance of the movement was designed and the actors involved in it.

The traffic accident management information system of the state of São Paulo (Infosiga SP) can be considered to be a legacy created by the movement for society. The system is a database that collects information from traffic accidents throughout the state. The information is updated monthly, which constitutes a major advance, and it provides fundamental support for the safety and agility of public policies that are aimed towards preventing and combating accidents, since other databases are only updated once a year.

By cross-referencing the information available in Infosiga SP, it is possible to obtain data on the locations of accidents, their causes and category, the victims’ age group and sex, vehicle specifications, the day and time, the most critical places and the aftermath of these accidents. Infosiga SP can make calculations at state, municipal and urban road levels, which makes it possible to create comparisons and cross-check the results in places where preventive policies have been implemented and in places where there have not been any actions targeting these issues.

The data facilitates better targeted actions, mitigation or elimination of risks and establishment of preventive policies and of protection for individuals with the most vulnerable profiles. The data also enable creation and improvement of processes and systems for urgent care of injured subjects, thereby avoiding harsher consequences and subsequent death or irreversible damage. They also provide support for decisions regarding strategies for traffic signaling and control.

**Figure 3.**
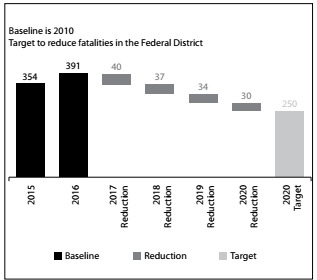
Target for reducing fatalities in the Federal District: reduce road fatalities by 50% by 2020, in line with the aims of the WHO road safety.

Infosiga SP integrates information from the 645 municipalities in the state of São Paulo. The system is fed with data from reports filed with the civil police, which are used to calculate traffic deaths. In addition, information from the military police and the federal highway police are used in formulating statistics on traffic accidents with victims. Figure 3 summarizes some of the information about the profile of accidents in the state of São Paulo. Out of the total number of recorded, 77% were men, 17% were women and 6% did not display this information.

### Safe Life Program of Brasília (Programa Brasília Vida Segura): staying ahead of WHO goals

Inspired by the success of the São Paulo Traffic Safety Movement, the Safe Life Program of Brasília (Programa Brasília Vida Segura) went even further and set more ambitious goals. The goal of this project was to reduce harmful use of alcohol by 10% by 2020. This challenge meant bringing forward the deadline set by WHO itself for the same rate to be reached around the world. In order to meet its goals, the Safe Life Program of Brasília was based on three factors: road safety, health and education. The first two (road safety and health) have been running since 2016 and have shown results that indicate that the model adopted is appropriate. The education factor was designed as a school and community-based preventive program to be implemented in 2019.

### Road safety: increasing life in traffic

**Figure 4.**
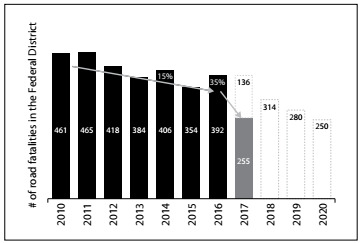
Road fatalities in the Federal District from 2010 to 2020: outstanding results from the first year of the project, almost reaching the target for the entire decade

The goal of the Safe Life Program of Brasília was to halve the number of lives lost every year in traffic accidents in the Federal District. The target was in line with the goals set by the United Nations for the decade of action for road safety. In terms of numbers, the Program predicts that, by 2020, road traffic deaths will be at a maximum of 250, whereas this number reached 392 in 2016. Figure 4 shows the trend as foreseen by the interventions conducted in Brasilia.

In order to better understand the traffic scenario in the Federal District, assess its risks and define the actions needed to eliminate them, the team of experts in charge of the Safe Life Program of Brasília undertook a comprehensive diagnosis. Their survey identified 230 actions that would be needed within the fields of engineering, education and control, following the same model that had already been adopted in São Paulo. It was also found that 34% of the fatalities in 2016 were related to alcohol consumption. Regarding age groups, the highest percentage of deaths was registered in the group of people between 30 and 39 years of age (34%), followed by the age ranges of 20 to 29 years and 40 to 49 years, both of which had a fatality rate of 21%. Pedestrians were the cohort that was most affected (34%), followed by drivers (29%). Regarding sex, the vast majority of the victims were men (89%).

All the effort to save lives was worthwhile. In 2017 alone, 133 lives were saved in traffic. This meant 34% fewer deaths (down to 255), compared with the total of 392 people who had lost their lives in traffic accidents in the Federal District in 2016. In other words, in just one year, the program almost reached the target set for 2020.

### Innovation in prevention of harmful use of alcohol

In addition to setting the target of decreasing hospitalizations caused by harmful use of alcohol by 10% by 2020, the Safe Life Program of Brasília has implemented a tool that was recommended by WHO for treating and managing cases, called brief intervention. This procedure is preceded by an alcohol use disorders identification test (AUDIT screening), which efficiently identifies the individuals who are at greater risk. Following this, the care protocol is defined according to the needs and conditions of each patient.

This intervention model was chosen based on studies and information that were obtained through official databases and then analyzed by specialists who were working in the program. Furthermore, the methodology recommended by WHO was completely concordant with the family health program (Programa Saúde da Família) and with the care provided at the primary healthcare units (Unidade Básica de Saúde, UBS) that the government maintains. Integrated work facilitates prevention because it enables identification and treatment of problems that are derived from harmful use of alcohol, at their initial stages.

In order to further elaborate the diagnoses upon which activities are based, the experts delved into databases that were made available by government bodies responsible for public health. This initiative allowed the professionals to identify and quantify the hospitalizations caused by harmful use of alcohol. Studying the information thus obtained allowed the researchers to have a better understanding of the scenario, both in relation to the causes for hospitalization and in relation to the patients’ profiles. Based on this assessment, they defined the group of men aged 40 to 49 years as the critical profile for the project, and pointed out the causes of the problem and its possible solutions.

With the aim of disseminating this new concept for preventing and combating harmful use of alcohol to the population, the Safe Life Program of Brasília trained 41 healthcare agents (doctors and nurses). These professionals received 15 hours of training and became able to put the protocols thus devised into practice. In total, 1,645 people living in the satellite towns of Ceilândia and Taguatinga, which were the locations chosen for beginning the implementation of the project, underwent screening and 77 of them were referred for treatment.

In only one year at work, the preliminary results from the Safe Life Program of Brasília indicated that the healthcare activities within the program were able to reduce the number of hospitalizations relating to harmful use of alcohol. To achieve the goals for reducing NCDs, great efforts towards implement the guidelines established by WHO have been made and, so far, within a brief period of time, there has been consistent progress.

### Education: preventing underage alcohol consumption

Prevention of underage alcohol consumption is the third goal of the project. A randomized survey was conducted in schools from April to May 2018 to understand the context of alcohol use among underage students from 13 to 17 years old in public and private schools.

The data obtained will help define specific actions aimed at reducing alcohol use among school students. Interventions will take place in public schools, but before they are disseminated in all the places benefited by the project, they will be tested in pilot areas. The results from the survey will provide a baseline for monitoring and managing the project. With these data, it will be possible to create a database, evaluate the effectiveness of the measures adopted and identify places or groups of people that need to be prioritized.

### Governance

The governance of the Safe Life Program of Brasília was based on a public-private partnership model, which allows for agile management and speedy results. The program is coordinated by a nationally based non-governmental organization (NGO) that focuses on developing leadership, activities and knowledge regarding public policies.

This NGO is at the heart of the organizational chart and its role is to integrate the other actors. Both the format and the governance of the Safe Life Program of Brasília make it possible to state that the project constitutes an innovative public-private partnership for preventing NCDs.

## DISCUSSION

The two projects described here (São Paulo Traffic Safety Movement and Safe Life Program of Brasília) have brought governments closer to the goals established by WHO for prevention and reduction of NCDs. Participation and mobilization of private sector efforts have ensured agility in the projects and have stimulated formation and strengthening of a results-driven culture. The transfers of knowledge and process management technology and the development of concrete action plans have helped governments meet the recommendations and objectives proposed by WHO.

Based on the knowledge obtained and the results achieved, it is possible to state that these programs are examples of innovative policies that aim to prevent and combat the risk factors involved in NCDs. The project management shared between the private sector, civil society and government has given rise to a sustainable public policy model that can be reproduced in other Brazilian states. Involvement of the road safety agencies, which work to combat traffic accidents in their various areas of activity, and the synergy achieved through formation and implementation of management committees, have been fundamental for the success of the project.

It is also important to highlight that one of the greatest defining features of the models presented here is that they both use and cross-reference information. Data that previously were scattered across various departments and sectors of government dealing with traffic, healthcare and surveillance have been analyzed in an integrated manner. The system enables anticipation of trends, for action in critical locations, for support of education and training campaigns, for structuring of emergency room care systems and for rehabilitation services and their care teams. Adoption of public policies based on the knowledge and results obtained from the two programs provides solid support for enabling Brazil to meet the sustainable development goals, i.e. to ensure healthy lives and promote wellbeing at all ages.

## CONCLUSIONS

Both programs are innovative public policies that deal with health issues caused by external agents that ultimately account for rapid increases in days lost due to disability. Prevention of external causes of deaths and injuries, such as traffic-related violence, strongly correlates with changes in habits and actions, especially excessive consumption of alcohol, and with NCDs in Brazil.

## References

[1] WHO Decade of Action for Road Safety 2011-2020: saving millions of lives. Who.int..

[2] WHO Global Conference on Noncommunicable Diseases: Enhancing polices coherence between different spheres of policy making that have a bearing on attaining SDG target 3.4 NCDs by 2030.

[3] Haagsma JA, Graetz N, Bolliger I (2016). The global burden of injury: incidence, mortality, disability-adjusted life years and time trends from the Global Burden of Disease study 2013. Inj Prev.

[4] AMBEV, FALCONI, CLP, TRANSIT Retrato da Segurança Viária 2017.

